# Severe Adenovirus Infection Associated with Hemophagocytic Lymphohistiocytosis

**DOI:** 10.4274/Tjh.2013.0218

**Published:** 2014-03-05

**Authors:** Ferda Özbay Hoşnut, Figen Özçay, Barış Malbora, Şamil Hızlı, Namık Özbek

**Affiliations:** 1 Başkent University Faculty of Medicine, Department of Pediatric Gastroenterology, Hepatology, and Nutrition, Ankara, Turkey; 2 Dr. Sami Ulus Research and Training Hospital of Women’s and Children’s Health and Diseases, Ankara, Turkey; 3 Dr. Sami Ulus Research and Training Hospital of Women’s and Children’s Health and Diseases, Department of Pediatric Gastroenterology, Hepatology, and Nutrition, Ankara, Turkey; 4 Başkent University Faculty of Medicine, Department of Pediatric Hematology, Ankara, Turkey

**Keywords:** Acquired hemophagocytic lymphohistiocytosis, Intravenous immunoglobulin, Adenovirus-associated hemophagocytic lymphohistiocytosis

## TO THE EDITOR

In healthy children adenoviral infection causes a benign, self-limited illness [1]. However, in immunocompromised patients, adenovirus can cause fulminant or disseminated disease such as colitis, pneumonitis, pancreatitis, nephritis, encephalitis, and hemophagocytic lymphohistiocytosis (HLH) [1,2]. Herein, we report the clinical course and the treatment of an infant who had no history of immune defects, familial HLH, or malignant disease and suffered from adenoviral pneumonia with progressive clinical deterioration due to onset of virus-associated HLH. In the literature there are few reports about adenovirus-associated HLH in healthy children. 

A previously healthy 11-month-old boy was admitted to a state hospital with fever and cough. His chest roentgenogram showed bilateral pneumonic infiltrates. The child was treated with ceftriaxone and amikacin but the pneumonia progressively worsened. Therefore, on the fifth day, his antibiotic therapy was changed to imipenem, amikacin, and vancomycin. The same day, a generalized myoclonic seizure was observed. The seizure was controlled by phenytoin. His liver function test results were elevated and the coagulation profile was deranged. Subsequently he was admitted to our hospital. 

On admission, he was confused. There was no response to verbal stimulus but he did respond to localized painful stimulus. His body temperature was 38.9 °C. He was tachypneic with retractions. Coarse crackles were audible in the right hemithorax, and in the left hemithorax, bronchial breathing was heard. The liver was enlarged 5 cm below the right subcostal margin. His spleen was 2 cm below the left subcostal margin. 

Complete blood count findings were as follows: hemoglobin, 7.1 g/dL; red blood cell distribution width, 18.3%; mean corpuscular volume, 68 fL; mean corpuscular hemoglobin, 18.9 pg; mean corpuscular hemoglobin concentration, 321.5 g/L; leukocyte count, 4.05x10^9^/L; absolute neutrophil count, 1.5x10^9^/L; and platelet count, 79.4x10^9^/L. Microcytic hypochromic anemia was shown in the peripheral blood smear examination. His reticulocyte count and vitamin B12 and folate levels were normal. His direct Coombs test results were negative. Fibrinogen levels were 209 mg/dL (reference range: 200-400). Biochemical findings revealed aspartate aminotransferase level of 641 U/L (reference range: 0-40), alanine aminotransferase of 219 U/L (reference range: 0-41), ferritin of 1820 ng/mL (reference level: 20), and triglyceride of 373 mg/dL (reference range: 35-110). Informed consent was obtained.

Chest computerized tomography demonstrated pleural effusion in the left hemithorax and bilateral consolidation. In diagnostic thoracentesis there were no visible pathologic findings. Serologies for chlamydial pneumonia, mycoplasma pneumonia, and respiratory syncytial virus were all negative. On the first day of admission, a localized myoclonic seizure was observed in his right arm. The patient required calcium-magnesium therapy because of his low levels of serum calcium and magnesium. Cerebrospinal fluid test showed that the protein and glucose levels were in the normal range with no pleocytosis. 

**Figure 1:**
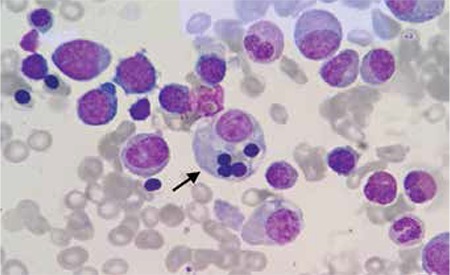
Wright staining of a bone marrow smear shows hemophagocytosis.

Bone marrow aspiration revealed increased numbers of histiocytes and hemophagocytosis (Figure 1). The serum study was positive for anti-adenovirus IgM and IgG. Adenovirus positivity was detected in the serum at 1.3x10^5^ copies/mL by a very sensitive, commercially available real-time PCR assay. These findings indicated that the patient had developed HLH, associated with primary adenovirus infection. 

Intravenous immunoglobulin (IVIG) was given for 2 days (0.5 g/kg/day). After the second dose of IVIG therapy, his general condition improved. Within 3 days, fever and hepatosplenomegaly disappeared and transaminase levels returned to the normal range. Complete blood count revealed hemoglobin of 10.2 g/dL, leukocyte count of 8.35x10^9^/L, absolute neutrophil count of 3.8x10^9^/L, and platelet count of 151.2x10^9^/L on day 21 of hospitalization. After 24 days, the patient was discharged without any problems. He is currently 27 months old and has no problems. On the tests done to enlighten the etiology of microcytosis, he was diagnosed as iron deficiency anemia. Ferrous sulphate therapy was started with a proper dosing. 

Although many viruses, such as the Epstein-Barr virus, human immunodeficiency virus, parvovirus, and hepatitis viruses, have been reported to cause infection-associated HLH, severe hemophagocytosis due to acute adenovirus infection is unusual [3]. There have been a limited number of case reports describing adenovirus pneumonia complicated with HLH [2,4,5,6]. In these reports, adenoviral pneumonia with HLH was successfully treated with IVIG and clarithromycin, dexamethasone and cyclosporine, or pulse methylprednisolone [2,4,6]. Antiviral treatment with cidofovir or ribavirin in adenovirus infections is being increasingly used, especially in immunocompromised patients; however, the efficacy of these drugs is not established [7,8].

We chose IVIG therapy for treatment of HLH in our patient. Because virus-associated HLH is rare, no certain treatment protocols have been described, and the role of IVIG in the treatment of HLH is unclear [3]. However, several studies have shown the beneficial effect of IVIG. Chen et al. [9] noted remission in only 2 of 9 children with virus-associated HLH treated with IVIG alone. Similarly, Goulder et al. [10] reported a 1-year-old boy with virus-associated HLH successfully treated with IVIG. In our patient, progressive deterioration was reversed into marked improvement after IVIG, suggesting the therapeutic benefit of this treatment. 

In our patient we were able to control infection-associated HLH with IVIG administration. We wanted to emphasize this good clinical and hematologic response. In conclusion, when a patient suffering from adenovirus is seen with prolonged fever unresponsive to antibiotics, hepatosplenomegaly, and cytopenias, HLH should be considered in the differential diagnosis. 

## CONFLICT OF INTEREST STATEMENT

The authors of this paper have no conflicts of interest, including specific financial interests, relationships, and/or affiliations relevant to the subject matter or materials included.
